# Review article: effects of type 2 diabetes therapies on bone metabolism

**DOI:** 10.1186/s13098-017-0274-5

**Published:** 2017-09-25

**Authors:** A. G. D. Vianna, C. P. Sanches, F. C. Barreto

**Affiliations:** 1 Curitiba Diabetes Center, Division of Endocrinology, Hospital Nossa Senhora das Graças, Rua Alcides Munhoz, 433–4° andar–Mercês, Curitiba, Paraná 80810-040 Brazil; 2 Pontifical Catholic University of Parana, Rua Imaculada Conceição, 1155–Bloco Medicina–Prado Velho, Curitiba, Paraná 80215-901 Brazil; 3 Division of Nephrology, Department of Internal Medicine, Federal University of Paraná, Rua General Carneiro, 181–Alto da Gloria, Curitiba, Paraná 80060-900 Brazil

**Keywords:** Type 2 diabetes, Bone metabolism, Fractures, Antidiabetic therapy, Treatment, Bone mineral density

## Abstract

Diabetes complications and osteoporotic fractures are two of the most important causes of morbidity and mortality in older patients, and they share many features, including genetic susceptibility, molecular mechanisms, and environmental factors. Type 2 diabetes mellitus (T2DM) compromises bone microarchitecture by inducing abnormal bone cell function and matrix structure with increased osteoblast apoptosis, diminished osteoblast differentiation, and enhanced osteoclast-mediated bone resorption. The linkage between these two chronic diseases creates a possibility that certain antidiabetic therapies may affect bone function. The treatment of T2DM has been improved in the past two decades with the development of new therapeutic drugs. Each class has a pathophysiologic target related to the regulation of the energy metabolism and insulin secretion. However, both glycemic homeostasis and bone homeostasis are under the control of common regulatory factors. This background allows the individual pharmacological targets of antidiabetic therapies to affect bone quality due to their indirect effects on bone cell differentiation and the bone remodeling process. With a greater number of diabetic patients and antidiabetic agents being launched, it is critical to highlight the consequences of this disease and its pharmacological agents on bone health and fracture risk. Currently, there is little scientific knowledge approaching the impact of most anti-diabetic treatments on bone quality and fracture risk. Thus, this review aims to explore the pros and cons of the available pharmacologic treatments for T2DM on bone mineral density and risk fractures in humans.

## Background

Patients with poorly controlled type 2 diabetes mellitus (T2DM) are at an increased risk of diabetic complications, including macrovascular disease, retinopathy, nephropathy, and neuropathy. Recently, an increased risk of fragility fractures has been recognized as another significant diabetes complication [1]. Two meta-analyses demonstrated that individuals with diabetes mellitus have an excessive risk of hip fractures, and this relationship is more pronounced in type 1 diabetes, although type 2 diabetes patients carried a 1.34 relative-risk compared with nondiabetic populations. The association between diabetes and hip fracture risk is similar in men and women [2, 3].

It has been observed that T2DM negatively affects the bone strength and the risk of fractures, regardless of bone mineral density (BMD) [4, 5]. The reasons involve likely a combination of features, including the duration of disease, inadequate glycemic control, a greater risk of falling as a consequence of hypoglycemia, osteopenia, an impairment of bone quality, and the side effects of medication, which could lead to a higher risk of bone fragility and fractures [5].

Bone quality is preserved by the process of bone remodeling, that comprises continuous resorption and formation of bone to substitute old tissue with new tissue. An equilibrium between osteoclast-dependent bone resorption and bone formation, the latter of which relies on osteoblast activity, is essential for the maintenance of bone mass [6]. Attenuated remodeling process characterizes bone in diabetes. Circulating levels of biochemical markers of bone formation and resorption are decreased in diabetes [7]. It is speculated that low turnover of bone in diabetes may lead to defective micro-fracture repairs and, hence, to their accumulation, contributing to decreased bone quality. In contrast to postmenopausal and senile osteoporosis, a deterioration of bone strength in diabetes is associated with increased cortical porosity that is not accompanied by a loss of trabecular bone mass [8, 9]. Thus, it can be concluded that diabetes-specific bone characteristics may constitute a novel syndrome that can be classified as a diabetes-associated bone disease. For more detail about bone biology on diabetes context, the authors suggest reading of the basic biology of diabetes, bone, and glucose lowering agents [6].

Moreover, an accompanying review about the impact of type 2 diabetes on bone metabolism review is provided in this issue of Diabetology & Metabolic Syndrome by Sanches CP, Vianna AGD and Barreto FC (Brief Review: The Impact of Type 2 Diabetes on Bone Metabolism).

Currently, there is little scientific knowledge approaching the impact of most anti-diabetic treatments on bone quality and fracture risk. Thus, this review aims to explore the pros and cons of the available pharmacologic treatments for T2DM on bone mineral density and risk fractures in humans. Type 1 diabetes mellitus will not be addressed in this text.

### Fracture risk assessment in diabetes

Fracture risk assessment offers specific challenges in the diabetic population. The commonly used assessment tools tend to miscalculate and underestimate risk in adults with diabetes [6].

Currently, BMD is considered the gold standard method for osteoporosis diagnosis by the World Health Organization (WHO), but its low sensitivity may result in loss of some diagnosis if used isolated. The BMD is expressed as a T-score, that is the number of standard deviations that the individual is above or below the average of a healthy adult [6].

The fracture risk assessment toll (FRAX) is largely used and incorporates femoral neck BMD T-score with additional risk factors: age, sex, BMI, history of fracture, parental history of hip fracture, current smoking status, alcohol consumption, rheumatoid arthritis and glucocorticoid intake. FRAX algorithm does not include diabetes as a risk factor. As a result, FRAX is unsatisfactory to estimate the fracture risk in diabetic patients [1]. The FRAX algorithm must be reviewed to include diabetes as a risk factor [10]. Currently, clinicians should be alert of the trend for FRAX to underestimate fracture risk in diabetes.

In the last decade, there has been considerable progress in the identification and characterization of specific biomarkers that help the management of osteometabolic disease, so-called bone turnover markers (BTMs) [11]. Recent recommendations of Bone Marker Standards Working Group proposed standardization for studies using a specific marker of bone resorption (CTX—carboxi-terminal telopeptide of type 1 collagen) and another specific one of bone formation (P1NP—amino-terminal propeptide of procollagen type 1) [12]. These markers, in combination with BMD, help to characterize better the evolution and conditions that interfere with the bone metabolism [13].

## Anti-hyperglycemic therapies and their effects on bone

T2DM is characterized by glucose intolerance and insulin resistance, which ultimately lead to hyperglycemia and hyperinsulinemia. Anti-hyperglycemic treatments comprise insulin sensitizers, insulin secretagogues, glucagon-like peptide (GLP)-1 receptor agonists, dipeptidyl peptidase-4 (DPP-4) inhibitors, sodium-glucose transporter (SGLT)2 inhibitors, and insulin. Each class of antidiabetics acts through distinct biological pathways, which may confer different drug class-specific potential benefits and disadvantages. Few high-quality studies have evaluated the bone effects of oral antidiabetics [14]. However, several pieces of evidence show that their effects on bone mineral density, the incidence of fractures and the markers of bone remodeling may be diverse, depending on the class of antidiabetics under examination. Henceforth, we will describe the effects of the most prescribed approved therapies for T2DM on bone.

### Metformin (Biguanide)

**Fig. 1:**
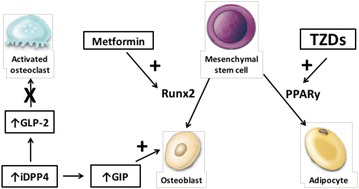
The potential effects of metformin, DPP4 inhibitors, and TZDs on bone metabolism. Metformin has a positive effect on osteoblast differentiation by activating the osteoblast-specific Runx2 transcription factor via the AMPK/USF-1/SHP regulatory cascade and an adverse effect on osteoclast differentiation by decreasing RANKL and increasing osteoprotegerin levels. PPARγ activation is associated with fewer osteoblasts, an increased number of adipocytes, greater support for osteoclastogenesis. DPP-4 inhibitors act to stabilize active forms of GIP and GLP-2. GIP increases osteoblast activity, and GLP-2 decreases osteoclast action. *iDPP4* dipeptidyl peptidase-4 inhibitor, *GIP* glucose-dependent insulinotropic peptide, *GLP*-*2* glucagon-like peptide-2, *TZDs* thiazolidinediones, *PPARγ* peroxisome proliferator-activated receptor-gamma, *Runx2* runt-related transcription factor 2. Modified from Gilbert et al. [26]

Metformin, the most studied biguanide, increases insulin sensitivity in T2DM patients. It acts by decreasing hepatic glucose production and increasing glucose uptake in muscle [15]. Metformin likely improves glucose metabolism via the activation of AMP-activated protein kinase (AMPK), which results in the suppression of fatty acid synthesis, the stimulation of fatty acid oxidation in the liver, an increase in muscle glucose uptake and a decrease in the expression of sterol regulatory element-binding-protein 1 (SREBP-1) [16]. SREBP-1 is involved in adipocyte differentiation and the pathogenesis of insulin resistance, dyslipidemia, and diabetes. The exact effect of AMPK on bone metabolism is not yet understood. Some reports suggest that AMPK modulates bone cell differentiation and function [17]. Results from in vitro and animal studies have shown that metformin has a positive effect on osteoblast differentiation (Fig. 1) by activating the osteoblast-specific Runx2 (runt-related transcription factor 2) transcription factor via the AMPK/USF-1/SHP regulatory cascade. Metformin also has an adverse effect on osteoclast differentiation by decreasing the pro-osteoclastic cytokine receptor activator of nuclear factor κB ligand (RANKL) and increasing osteoprotegerin levels [18–20]. Metformin could enhance bone mineral content, bone mineral density, and percent bone volume, and decrease trabecular separation in ovariectomized rats, indicating the attenuation effect of metformin on bone loss induced by ovariectomy [20].

The ADOPT study (A Diabetes Outcome Progression Trial) did not demonstrate a beneficial effect of metformin on fracture risk [21]. However, metformin decreased levels of the serum marker of bone resorption C-terminal telopeptide of type I collagen (CTX) and the serum formation marker PINP (amino-terminal propeptide of procollagen type 1) [22]. A recent investigation indicated that after an 80-week treatment, the combined therapy of rosiglitazone plus metformin was associated with significantly reduced BMD in lumbar spine and hip, while metformin monotherapy did not affect bone mass [23]. Further randomized placebo-controlled studies are required to evaluate the effects of metformin on bone metabolism. Available data support the hypothesis that metformin has a neutral effect on BMD and fracture risk.

### Thiazolidinediones (Rosiglitazone and Pioglitazone)

Thiazolidinediones (TZDs) increase insulin sensitivity through the activation of peroxisome proliferator-activated receptor-gamma (PPARγ) [24]. They are excellent therapeutic approach for treating T2DM, but their prolonged use promote some adverse effects, such as fluid retention and weight gain [13]. Clinical evidence suggests that these drugs cause bone loss and can increase fracture risk [21, 25–27].

The risk factors related to increased fractures in TZD users are female gender, increasing age, pre-existing conditions (comorbidities, corticosteroid use, smoking, and history of previous fracture) and the duration of treatment, as will be reviewed subsequently [23]. Changes in BMD have been accompanied by a modification in bone turnover markers. Rosiglitazone therapy has been associated with a reduction in the markers of bone formation, such as bone-specific alkaline phosphatase (BALP) and PINP, and a significant increase in the levels of the resorption marker CTX in women, but not in men [22]. Nevertheless, both genders have decreased levels of PINP. The rise in bone resorption markers in female patients may explain the increased fracture rate in this gender with TZD therapy [22]. The fracture risk further increases with the duration of treatment, and pioglitazone is more strongly associated with fractures than rosiglitazone, especially in men [28]. An additional observational study based on the United Kingdom General Practice Research Database (GPRD) showed that TZD therapy and the duration of treatment are associated with a significant increase in nonvertebral fractures, independent of patient sex and age [29]. Furthermore, a self-controlled case-series study on the GPRD population strongly suggested that prior fracture also contributes to raising the risk of the next fracture occurrence [30].

Several studies propose that the effects of TZD on bone are a drug-class effect. Women and elderly are at an increased risk of bone loss and fractures, especially those who have a history of prior TZD-unrelated fractures [23]. The explanation for the TZD-induced bone loss is demonstrated by the mechanism of PPARγ, which is a target of these antihyperglycemic agents. As described previously, the activation of the PPARγ2 protein by rosiglitazone on bone tissue determines the permanent conversion of osteoblastic cells to adipocytes and, additionally, suppress the particular genetic expression of osteoblasts and their phenotype (Fig. 1) [25]. This concept is supported by in vitro studies and suggests that PPARγ2 is a positive regulator of adipocyte differentiation and acts as a dominant-negative regulator of osteoblast differentiation [25, 31].

Findings in animal models have confirmed the role of PPARγ in the maintenance of bone homeostasis, depending on the status of PPARγ activity [32–34]. PPARγ activation is associated with a decreased number of osteoblasts, an increased number of adipocytes, greater support for osteoclastogenesis and, ultimately, bone loss. Younger animals exhibited bone loss due to decreased formation; however, in older animals, it was due to increased resorption [34]. Analysis of gene expression in mesenchymal stem cell (MSC) of animal models displayed reduced expression of essential genes that control bone homeostasis in animal models treated with rosiglitazone [35, 36]. The collection of evidence suggests that TZDs cause bone loss and increase fracture risk.

### Sulfonylureas

Sulfonylureas act as insulin secretagogues by binding to receptors on the pancreatic β-cell surface (sulphonylurea receptor, SUR1) and consequently stimulating the exocytosis of insulin [37]. These drugs have been used to treat long-standing diabetic patients for more than 50 years, but little clinical data exist about this therapy on bone health. Because of the association between sulfonylurea and hypoglycemia, it is reasonable to link its use to falls and fractures. Studies attempting to quantify the association between sulfonylureas and fall-associated fractures have yielded contradictory results [38]. This interconnection occurs only in elderly subjects with moderate limitations in activities of daily living. Moreover, the reasons for falling are multifactorial. The fraction of falls attributable to a sulfonylurea use may be relatively small compared to the falls caused by other frequently used medications, the independent effects of multiple comorbidities, and environmental factors. Again, evidence from both the ADOPT study and the Rochester study indicate that glyburide, one of the class representatives, does not have an effect on bone metabolism and fracture risk [21, 39], with exception of the decreased serum levels of the bone formation marker PINP with glyburide therapy [22]. A recent study published by our group suggests that gliclazide modified release, a sulfonylurea, does not change bone markers concentrations nor BMD [40]. The existing data support the impression that sulfonylureas have a minimal effect and are at least neutral concerning BMD and their effects regarding fractures are confounded by the hypoglycemia-induced fall risk in elderly subjects.

### GLP-1 receptor agonists

Glucose-dependent insulinotropic peptide (GIP) and glucagon-like peptides 1 and 2 (GLP-1 and GLP-2) are hormones released by gut enteroendocrine K-cells in the duodenum and proximal jejunum and from L-cells located in the distal ileum and colon, respectively [41]. The incretin hormones (GIP and GLP-1) are secreted just after nutrient ingestion, and they stimulate insulin release from β-cells and inhibit glucagon production by α-cells [42]. The role of incretin hormones in bone turnover is associated with nutritional status. In addition to their action in enhancing insulin and suppressing glucagon secretion in response to glucose intake, there is evidence that the incretin hormones play an essential role in bone homeostasis [43].

Studies have established that GIP receptors are present on osteoblast and osteoclasts. The former is responsible for the activation of those receptors, increasing collagen type 1 synthesis and alkaline phosphatase activity, which is consistent with an anabolic effect. In osteoclasts, GIP might inhibit resorptive activity, and it inhibits the expression of some markers of osteoclastic differentiation [44].

Recently, it has been revealed that GLP-1 receptors are expressed on osteoblasts, inducing an anabolic effect on bone remodeling [45], whereas receptors for GLP-2 were evidenced in osteoclasts, acting through the reduction of bone absorption [43].

**Fig. 2:**
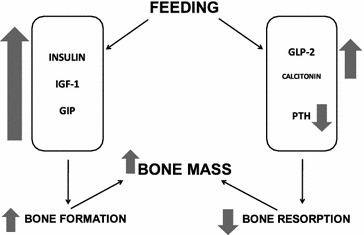
The proposed relation between nutrition and bone mass. GIP, and possibly GLP-1 and GLP-2, may link nutrient ingestion to the suppression of bone resorption and the stimulation of bone formation. This link is likely because both osteoblasts and osteoclasts express receptors for incretins, which positively regulate bone metabolism *IGF*-*1* insulin-like growth factor-1, *GIP* glucose-dependent insulinotropic peptide, *GLP*-*2* glucagon-like peptide-2, *PTH* parathyroid hormone. Modified from Reid et al. [47]

In periods of energy and nutrient overload, the balance is shifted towards bone formation, whereas bone resorption increases in energy and nutrient insufficiency [43]. GIP, and possibly GLP-1 and GLP-2, may link nutrient ingestion to the suppression of bone resorption and the stimulation of bone formation. This link is likely because both osteoblasts and osteoclasts express receptors for incretins, which positively regulate bone metabolism (Fig. 2) [46].

Wnt/β-catenin signaling is involved in this process, mainly because sclerostin, a protein produced by mature osteocytes, is inhibited by GLP-1 receptor agonists [48]. Moreover, thyroid C-cells express GLP-1 receptors, and their activation induces calcitonin release and thus indirectly inhibits bone resorption [48]. Altogether, they result in an increase in intracellular calcium levels, alkaline phosphatase activity, and collagen type 1 expression, which ultimately improves bone mass [49].

A significant number of in vitro and animal studies support the potential role of intestinal hormones in modulating bone metabolism. They indicated that GLP-2 acts as an antiresorptive hormone, while GIP can act as both an antiresorptive and an anabolic hormone on bone by protecting osteoblasts against apoptosis [44]. GIP receptor (GIPR) knockout mice (GIPR-/-) exhibit low BMD secondary to increased bone resorption and decreased bone formation, along with reduced bone mass and altered microarchitecture. Moreover, knockout animals have reduced levels of serum markers of bone formation, including osteocalcin and alkaline phosphatase [50]. However, in humans, there are limited data about the effect of GIP on bone metabolism.

The biological effect of GLP-1 on bone has been studied in animals and has been observed in humans. Yamada et al. showed that knock-out mice for the GLP-1 receptor had osteopenia and an increase in bone fragility, due to increased bone resorption. Moreover, an in vitro analysis suggests that GLP-1 does not appear to have a direct effect on osteoblasts or osteoclasts, but rather, it acts through calcitonin to modulate bone turnover. The survey summary indicates that GLP-1 receptor signaling indirectly participates in the control of bone resorption through a calcitonin-dependent pathway [51].

In humans, GLP-1 receptor agonists are an alternative approach for obtaining the beneficial effects of the incretin system in the treatment of T2DM. Previous animal studies suggested that GLP-1 agonists could impact bone metabolism by mimicking the physiological actions of native GLP-1, affecting the fat-bone axis by promoting osteogenic differentiation and inhibiting adipogenic differentiation of bone MSCs. This effect may influence the balance between osteoclasts and osteoblasts, thus leading to increased bone formation and decreased bone resorption [52]. Few clinical trials have addressed the impact of GLP-1 receptor agonists on bone metabolism.

Liraglutide, a GLP-1 receptor agonist (GLP-1RA), was capable of improving trabecular volume and thickness and the number of trabeculae, reducing trabecular space and increasing BMD in rats, as demonstrated by micro-CT analysis [52]. Forty-four-week treatment with exenatide, another GLP-1RA, had no effect on BMD in 36 patients with T2DM [53]. Li et al. established that a 24-week treatment with exenatide had no impact on bone turnover markers or BMD [54]. Recent trials and meta-analyses reported a lack of available data to determine whether there was an altered risk of fracture in patients treated with a GLP-1 agonist [25]. Another recent meta-analysis demonstrated a conflicting information about the risk of bone fractures associated with different GLP-1 RA treatments, with an odds ratio (OR) of 0.38 favorable to reduced risk of fractures with liraglutide treatment, and an OR 2.09 showing an elevated risk of incident bone fractures with exenatide [55]. Driessen et al. performed population-based cohort and case–control studies and concluded that there was no decreased risk of fracture with current use of GLP1-RA compared to never- GLP1-RA use. Osteoporotic fracture risk was also not reduced by current GLP1-RA use [56, 57]. The current findings need to be confirmed by future well-designed prospective or RCT studies.

Since GLP-1RA therapy is relatively new, more knowledge is necessary to establish whether the properties of these drugs may contribute to decreased bone fragility in diabetic patients. The currently available data do not permit to evaluate the effect of GLP-1 receptor agonist on BMD and risk of fractures.

### DPP-4 inhibitors

DPP-4 inhibitors (iDPP-4) represent one of the newest classes of oral anti-diabetics. Sustained inhibition of DPP-4 lowers glycemia through increased insulin secretion and the inhibition of glucagon release [58]. They increase the serum concentration of incretin hormones by inhibiting the inactivation of endogenous GLP-1 and GIP by DPP-4.

We formerly defined that bone resorption is known to be inhibited by the acute nutrient ingestion mediated by incretins. DPP-4 inhibitors act to stabilize active forms of GIP, GLP-1, and GLP-2, improving postprandial calcium accretion, which thereby may have beneficial effects on bone health. GIP receptors are expressed in osteoblasts, and they increase osteoblast activity and protect osteoblasts from apoptosis (Fig. 1). In osteoclasts, GIP has been shown to inhibit parathyroid hormone (PTH)-induced bone resorption [50].

GLP-2 is thought to have a primary role maintaining the integrity of the gut epithelium. It is involved in the regulation of absorption and the disposal of nutrients in the postprandial period [59]. Some small studies in humans have inferred that GLP-2 may also play a role in inhibiting bone resorption after nutrient ingestion [60, 61], once osteoclasts have expressed their receptors [62] (Fig. 1). A sharp and sustained reduction in the markers of bone resorption (CTX) was noted after a 14-day treatment with a GLP-2 injection (subcutaneous, once daily) in 60 postmenopausal women [61]. To investigate the effect of GLP-2 on bone turnover, a dose escalation study on the subcutaneous injection of GLP-2 in healthy fasting postmenopausal women was conducted to assess the incretin effects by CTX. The results showed a significant dose-dependent reduction of serum CTX compared to the fasting individuals with placebo [62]. An examination of the markers of bone remodeling in these studies indicates that GLP-2 injections have no effect on the markers of bone formation, but they reduce bone resorption [60, 63].

On the other hand, thyroid C cells express GLP-1 receptors, and their activation stimulates the secretion of calcitonin, a potent inhibitor of osteoclastic bone resorption [64, 65]. This pathway may contribute to the nutrient-mediated reduction of bone resorption.

Female mice treated with sitagliptin, an iDPP-4, exhibited significant improvements in vertebral BMD and trabecular architecture [66]. Bunck et al. conducted a study to assess whether vildagliptin, an iDPP-4, had an impact on bone resorption and calcium homeostasis [67]. The authors concluded that treatment with vildagliptin 100 mg once daily was not associated with alterations in the markers of bone resorption (serum CTX) and calcium homeostasis in drug-naive patients with T2DM and mild hyperglycemia. A meta-analysis that evaluated the incidence of bone fractures in 11,880 patients on iDPP-4 and 9175 comparators revealed an odds-ratio of 0.60 (95% CI 0.37–0.99), which favored a protective effect of iDPP-4 on bone [68]. Conversely, a retrospective population-based cohort study (N = 216, 816) demonstrated no difference in the risk of fracture when iDPP-4 users were compared to controls [69]. Furthermore, a more recent meta-analysis (N = 62,206) showed that iDPP-4 does not seem to affect the risk of fracture when compared to placebo or other antidiabetic medications [58]. We previously reported a prospective trial comparing the bone effects of vildagliptin and gliclazide modified release (MR), a sulphonylurea, in a subset of postmenopausal women with T2DM. Neither drugs showed any positive or negative effects on the markers of bone formation or resorption or on BMD [40].

Since incretin therapy is relatively new, clinical data on its safety or potential benefits to bone is just emerging, and more studies are necessary to assign the effects of DPP-4 inhibitors on BMD and fracture risk. Existing data support that DPP-4 inhibitors do not change BMD and do not increase or decrease the risk of fractures.

### Sodium-glucose co-transporter 2 (SGLT2) inhibitors

SGLT2 inhibitors are currently the newest class of oral anti-diabetics. SGLT2 channels are nearly exclusively expressed in the proximal tubules of the kidneys and are responsible for 90% of renal glucose resorption [70]. SGLT2 inhibitors act independently of insulin and promote a negative energy balance through augmented glycosuria [71].

The SGLT-2 inhibitor canagliflozin, at least in one isolated analysis, appears to be associated with an enhanced risk of fractures, although the reason is still unclear [72, 73]. One possible explanation is that there is a higher incidence of fall, due to an increased risk of hypoglycemia associated with hypoglycemia-prone drugs, such as sulfonylureas or insulin. The risk of fall may also be increased by the mild volume depletion caused by these drugs. An increase in symptoms related to diabetic neuropathy or orthostatic hypotension may also be induced by the lower blood volume.

SGLT-2 inhibitors could also lead to a poorer bone mechanical quality. It is well established that they induce weight loss, which may predispose patients to a reduction in BMD [74]. To date, there is no evidence of SGLT-2 expression in bone. However, the inhibition of SGLT-2 could indirectly affect bone metabolism through the modulation of calcium/phosphate homeostasis. This modulation leads to an increased tubular reabsorption of phosphate, and increased serum phosphate levels are capable of stimulating parathyroid hormone secretion, which ultimately enhances bone resorption and the risk of fractures [75].

Additionally, SGLT-2 inhibitors could interfere with calcium metabolism by binding to the SGLT-1 receptor, albeit they have a very low affinity for this receptor. SGLT-2 inhibitors have a weak inhibitory activity on SGLT-1, which, in rodents, resulted in intestinal carbohydrate malabsorption and was accompanied by an enhancement of calcium absorption, a reduction in parathyroid hormone and 1,25-dihydroxy-vitamin D levels, hyperostosis, and hypercalciuria [76].

Previous studies indicated that canagliflozin might increase bone turnover, with increases in the biomarkers for both bone resorption (CTX) and bone formation (osteocalcin) in the serum [76], while dapagliflozin had no meaningful effect on the markers of bone turnover [77, 78]. Furthermore, a decrease in total hip BMD was detected in patients with T2DM using canagliflozin [77, 79]. Once again, dapagliflozin appeared to have no effect on BMD [77]. Ptaszynska et al. reviewed the safety of dapagliflozin in a pooled analysis of data from 12 placebo-controlled studies of a phase IIb/III trial and demonstrated no evidence of increased fracture risk [80, 81]. One pooled analysis of data from more than 11,000 patients with T2DM reported that empagliflozin was not related to an increased risk of bone fractures versus placebo [82]. Otherwise, a pooled analysis of 10 trials showed that canagliflozin only increased fracture risk in patients who were older, at a higher risk of cardiovascular disease, and had renal dysfunction or a greater baseline diuretic use [57].

In a recent meta-analysis of 38 randomized clinical trials with 496 fracture events among 30,384 patients with T2DM followed for 24–160 weeks, no significant difference in fracture risk was observed between SGLT2 inhibitor users and controls (OR 1.02; 95% CI 0.84–1.23). Moreover, there was no proof that a specific SGLT2 inhibitor (e.g., dapagliflozin, canagliflozin or empagliflozin) augmented the frequency of fracture or had distinct effects on bone. The authors suggested that the increased fracture rate associated with canagliflozin in one study might be attributable to chance or possibly other risk factors, even in subgroups of patients with an increased risk of fracture [83].

The currently available data from clinical studies and meta-analysis suggest that SGLT-2 inhibitor treatment was, in general, not associated with changes in bone mineral metabolism and BMD. Discrepant results from one study with canagliflozin may not yet attribute an increased risk to bone health to this agent or the whole class.

### Insulin

Insulin exhibits anabolic bone effects by binding to the insulin receptor expressed on osteoblasts, which through insulin receptor substrate (IRS-1 and IRS-2) stimulates bone formation by increasing proliferation and function.

Insulin deficiency is associated with skeletal defects that are explained by diminished linear bone growth during the pubertal growth spurt, reduced adult bone density, an augmented risk of fragility fracture, and poorer bone regeneration characteristics. This is supported by the frequent finding of osteopenia and osteoporosis among type 1 diabetes mellitus (T1DM) patients and supports the concept that insulin has anabolic actions on bone [84].

It is well known that T2DM, hyperinsulinemia, and insulin resistance are associated with increased bone density, but decreased bone strength, which contributes to an increased risk of fracture. Osteoblasts and their cell lineages express insulin receptors on the cell surface and have a high capacity for insulin binding [84]. In animal models, IRS-1 knockout mice have impaired bone healing, whereas IRS-2 knockout mice demonstrated lower indices of bone formation and evidence of enhanced bone resorption [85, 86].

In vivo, intensive insulin treatment seems to be neutral to BMD in T1DM patients over a 7-year period of follow-up. It is hard to distinguish whether the stability of BMD is due to the action of insulin or improved glycemic control [87]. A review of animal studies further supported the direct anabolic effect of insulin on bone, and it concluded that bone regeneration is impaired in insulin deficiency and can be restored by treatment with exogenous insulin [84].

Several studies attempt to elucidate the bone effect of insulin treatment in T2DM patients, but there are no randomized controlled trials designed to evaluate the impact of insulin on bone integrity in patients with T2DM. The Study of Osteoporotic Fractures compared the risk of fracture in postmenopausal women with T2DM who were managed with and without an insulin analog. An increased risk of foot fracture in the insulin-treated group [relative risk (RR) 2.54; 95% confidence interval (CI) 1.01–6.34] was demonstrated, but there was no higher risk of fracture at other skeletal sites [88]. Otherwise, Nicodemus et al. who also studied postmenopausal women with T2DM, showed that women who used an insulin analog were at the highest risk (RR 2.66; 95% CI 1.52–4.64) for a hip fracture [89].

Despite the anabolic role of insulin in bone, studies are controversial and inconsistent about the impact of insulin therapy on bone metabolism [84]. Insulin seems to increase BMD, but it can increase hypoglycemia-associated fall risk and it’s commonly used in the advanced phase of T2DM, increasing the fracture risk. The lack of randomized controlled trials regarding that effect makes it difficult to draw absolute conclusions.

### Summary

Table 1 summarizes the known effects of antidiabetic therapies on bone metabolism, BMD and the risk of fracture and Table 2 analyses the core details of each study cited in Table 1.

**Table 1:** Summary of the effects of antidiabetic drugs on bone metabolism

	Bone markers		Bone mineral density	Risk of fractures
	Resorption	Formation	(BMD)	(RF)
Metformin	↑ [22]	↓ [22]	↔ [90]	↔ [21]
Thiazolidinediones	↑ [22]	↓ [22]	↓ [22, 26]	↑↑ [21]
Sulfonylureas	↔ [22]	↓ [22]	↔ [21, 39]	Conflicting results [21, 39]
GLP-1 RA	↓ [48]	↑ [45]	NA	NA
iDPP-4	↔ [67]	NA	↔[40]	↔ [58, 69]↓ [68]
iSGLT2	↑ [76]	↑ [76]	↓[73]^a^ ↔ [77, 83]	↑ [72, 79]^a^ ↔ [77, 78, 83]
Insulin	NA	NA	↑	↑ [89, 91]

*↑* increases, *↓* decreases, *↔* neutral effect, *NA* data not available or insufficient evidence, *BMD* bone mineral density, *RF* risk of fractures

^a^Results for canagliflozin only

**Table 2:** Core details of the references mentioned in the analysis of BMD and RF in Table 1

References	Study category	Time of therapy before analysis (months) mean or range	HbA1c of the population at the baseline (%) Mean or range	Age (years) mean or range	Gender (M/F)
[21]	RCT	48	7.4	56	M/F
[22]	RCT	12	7.4	57	M/F
[26]	LC	48	8.4	73	M/F
[39]	LC	NA	NA	62	M/F
[40]	RCT	12	7.3	63	F
[58]	MA	3–48	6.7–9.9	50–75	M/F
[67]	RCT	12	6.0	57	M/F
[68]	MA	6–24	6.7–9.9	50–72	M/F
[69]	LC	12	8.3	59	M/F
[72]	RCT	29	8.2	62	M/F
[73]	RCT	43	8.2	63	M/F
[77]	RCT	24	6.5–8.5	61	M/F
[78]	RCT	11	7.2	61	M/F
[79]	RCT	24	7.7	64	M/F
[83]	MA	6–37	NA	NA	M/F
[89]	LC	114	NA	61	F
[90]	RCT	18	8.6	51	M/F
[91]	CC	49	8.0	70	M/F

*LC* longitudinal cohort, *CC* case–control study, *RCT* randomized controlled study*, MA* meta-analysis of RCT*, NA* data not available, *HbA1c* glycated haemoglobin, *M,* male*, F* female

## Conclusions

Patients with T2DM have an increased risk of fragility fractures, which was not predictable by BMD measurements. This higher risk is probably multifactorial, and antidiabetic therapies may have an impact on bone metabolism. The assessment of any drug effect on fracture risk is challenging because a beneficial or unfavorable action may become evident only after prolonged exposure. From a bone perspective, metformin and sulphonylureas are safer than TZDs. Physicians should use TZDs with caution in older diabetic subjects, who are at a greater risk of falls and fractures, particularly postmenopausal women. The newest antidiabetic therapies, such as GLP-1RA, DPP4 inhibitors, and SGLT2 inhibitors, seem to be harmless to the bone, but more studies are required to clarify their safety. The absence of randomized controlled trials makes it difficult form conclusions about the effect of insulin treatment on bone in T2DM patients.

## Abbreviations

ADOPT: a diabetes outcome progression trial
AMPK: AMP-activated protein kinase
BALP: bone-specific alkaline phosphatase
BMD: bone mineral density
CI: confidence interval
CTX: carboxy-terminal telopeptide of type 1 collagen
DPP-4: dipeptidyl peptidase-4
GIP: glucose-dependent insulinotropic peptide
GIPR: GIP receptor
GLP-1: glucagon-like peptide-1
GLP-1RA: GLP-1 receptor agonist
GLP-2: glucagon-like peptide-2
GPRD: general practice research database
iDPP-4: dipeptidyl peptidase-4 inhibitors
IRS-1: insulin receptor substrate-1
IRS-2: insulin receptor substrate-2
MR: modified release
MSC: mesenchymal stem cell
PINP: amino-terminal propeptide of procollagen type I
PPARγ: peroxisome proliferator-activated receptor gamma
PTH: parathyroid hormone
RR: relative risk
Runx2: runt-related transcription factor 2
SGLT1: sodium-glucose co-transporter-1
SGLT2: sodium-glucose co-transporter-2
SREBP-1: sterol regulatory element-binding-protein 1
SUR1: sulphonylurea receptor
T1DM: type 1 diabetes mellitus
T2DM: type 2 diabetes mellitus
TZDs: thiazolidinediones
